# The cost of community outreach HIV interventions: a case study in Thailand

**DOI:** 10.1186/s12889-021-12416-x

**Published:** 2022-01-06

**Authors:** Kyaw Min Soe, Katharina Hauck, Sukhum Jiamton, Sukhontha Kongsin

**Affiliations:** 1 Research Centre for Health Economics and Evaluation, Faculty of Public Health, Mahidol University, 420/1 Ratchawithi Rd, Khet Ratchathewi, Bangkok, 10400 Thailand; 2 Department of Infectious Disease Epidemiology, Faculty of Medicine, School of Public Health, Imperial College London, London, UK; 3 Department of Dermatology, Faculty of Medicine Siriraj Hospital, Mahidol University, Bangkok, Thailand

**Keywords:** Cost, Unit cost, Community-based, HIV intervention, Thailand

## Abstract

**Background:**

There was an estimated 440,000 people living with HIV in Thailand in 2018. New cases are declining rapidly thanks to successful prevention programs and scaling up of anti-retroviral therapy (ART). Thailand aims to achieve its commitment to end the HIV epidemic by 2030 and implemented a cascade of HIV interventions through the Reach-Recruit-Test-Treat-Retain (RRTTR) program.

**Methods:**

This study focused on community outreach HIV interventions implemented by Non-Governmental Organizations (NGOs) under the RRTTR program in 27 provinces. We calculated unit cost per person reached for HIV interventions targeted at key-affected populations (KAPs) including men who have sex with men/ transgender (MSM/TG), male sex workers (MSW), female sex workers (FSW), people who inject drugs (PWID) and migrants (MW). We studied program key outputs, costs, and unit costs in variations across different HIV interventions and geographic locations in Thailand. We used these estimates to determine costs of HIV interventions and evaluate economies of scale.

**Results:**

The interventions for migrants in Samut Sakhon was the least costly with a unit cost of 21.6 USD per person to receive services, followed by interventions for migrants in Samut Prakan 23.2 USD per person reached, MSM/TG in Pratum Thani 26.5USD per person reached, MSM/TG in Nonthaburi 26.6 USD per person reached and, MSM/TG in Chon Buri with 26.7 USD per person. The interventions yielded higher efficiency in large metropolitan and surrounding provinces. Harm reduction programs were the costliest compare with other interventions. There was association between unit cost and scale of among interventions indicating the presence of economies scale. Implementing HIV and TB interventions jointly increased efficiency for both cases.

**Conclusion:**

This study suggested that unit cost of community outreach HIV and TB interventions led by CSOs will decrease as they are scaled up. Further studies are suggested to follow up with these ongoing interventions for identifying potential contextual factors to improve efficiency of HIV prevention services in Thailand.

## Background

It was estimated that 480,000 people were living with HIV in Thailand in 2018 and about 15,000 people died from AIDS-related illnesses [1]. Nevertheless, new cases reported have been rapidly declining thanks to its successful prevention programs along with the scaling up of anti-retroviral therapy (ART) [1, 2]. With the aim to achieve its commitment to end the AIDS epidemic by 2030, Thailand has implemented a cascade of HIV interventions through Reach-Recruit-Test-Treat-Retain (RRTTR) programs to address the gaps in HIV prevention and life-long treatment system [2].

**Fig. 1:**
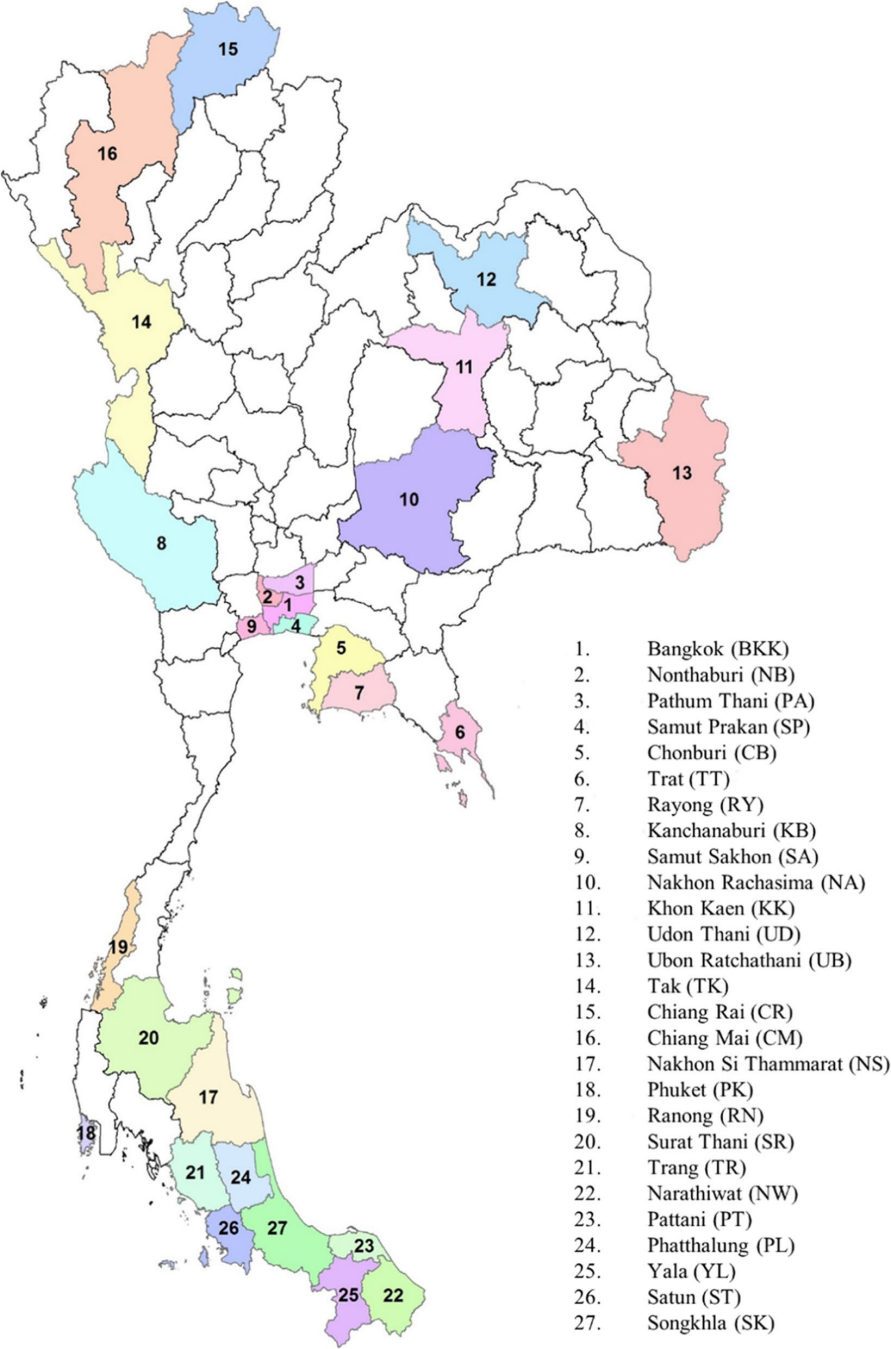
Provinces of community outreach HIV and TB interventions in Thailand

Domestic resources account for more than 85% of the policy response to HIV and TB in Thailand. Although international funding for HIV is a small fraction of overall funding, it is the main source of funding for HIV interventions targeting migrants and key-affected populations (KAPs). Most of domestic funding is focused on treatment and care [3, 4]. In 2015, Thailand launched a series of HIV preventive interventions through the Reach-Recruit-Test-Treat-Retain (RRTTR) program with the support of the Global Fund. The strategic short-term plan of RRTTR is to prepare the country for scaling down of Global Fund investment and facilitate the transition of HIV financing to domestic sources. Under RRTTR, civil society organizations (CSOs) or non-governmental organizations (NGOs) were contracted as implementing agencies (IAs) for community-based preventive interventions in an active case finding approach to reach out to KAPs, improve uptake of for HIV counselling and testing (HCT) and support effective linkages to treatment initiation and retention through collaboration with government health service providers [5]. A total of 38 community outreach HIV interventions covering the Reach and Recruit components of RRTTR for different KAPs were implemented in 27 provinces (Fig. 1) with high burden of HIV and TB. RRTTR also granted some IAs to provide TB care services for migrant workers in combination with HIV services [6].

A formal empirical evaluation of the RRTTR program has not yet been undertaken. It is believed that efficiency gains or cost reductions could be achieved through economies of scale and scope. Economies of scale are reductions in unit cost of a service that might be achieved when the scale or volume of that service’s provision is increased. In the other hand, economies of scope are the reductions in unit cost of a service that might be observed when that service is provided jointly or in combination with other services [7–9]. This study aimed at finding evidence for Economies of Scope and Scale. We calculated unit costs per person of HIV interventions for men who have sex with men (MSM) and transgender people (TG), male sex workers (MSW), female sex workers (FSW), people who inject drugs (PWID), and migrant workers (MW) at the community level which were implemented under this program. We used these estimates to determine the cost of interventions and evaluate the economies of scale and scope. The results from this study aimed to provide strategic information for policy makers on setting priorities and optimizing efficiency in resources used for HIV programs within a sustainability financing mechanism when scaling-up.

## Methodology

### Research setting

This study focused on community-based HIV interventions implemented by Non-Governmental Organizations (NGOs) as Implementing agencies (IAs) under RRTTR program in 27 provinces with relatively high prevalence of HIV in Thailand (Fig. 1) [6]. The authors examined the costs of 38 HIV interventions and their outputs in terms of the number of key affected populations (KAPs) who received HIV services, i.e. who were reached and recruited into the care system. There was a total of 20 IAs involved in this study (Table 1) and data were collected from IAs routine reports covering the reporting period from January 2015 to September 2016. The data used in the study was de-identified or anonymized for confidentiality and protecting privacy of study participants.

**Table 1:** Characteristics of community outreach HIV interventions in this study

Characteristics	Number	%
**HIV interventions**		
No. of implementing agencies (IAs)	20	
No. of provinces covered	27	
No. of community outreach HIV interventions	38	
Total cost spent by IAs for HIV interventions (USD)	7,837,234	100.0
- Direct program cost by IAs at site level (USD)	5,576,092	71.1
- Indirect program cost or overhead cost (USD)	2,261,142	28.9
- Mean Cost	146,739	
- Min. Cost	31,122	
- Max. Cost	294,562	
- Standard Deviation	104,474	
No. of KAPs **reached** by HIV interventions (person)		
- Total No. of persons **reached**	190,931	
- Mean	5025	
- Minimum	161	
- Maximum	25,438	
- Standard Deviation	5367	
No. of KAPs received HIV tests (person)		
- Total no. of KAPs **tested**	32,309	
- Mean	850	
- Minimum	20	
- Maximum	1548	
- Standard deviation	936	
**TB interventions**^a^		
▪ No. of implementing agencies (IAs)	4	
▪ No. of provinces covered	7	
▪ No. of community outreach TB interventions	7	
▪ Cost spent by IAs for TB interventions (USD)	1,636,082	100.0
▪ Direct program cost by IAs at site level (USD)	1,110,065	67.8
▪ Indirect program cost or overhead cost (USD)	526,017	22.2
▪ No. of migrant population reached (person)	56,099	
▪ No. of TB suspects identified (person)	878	

^a^All TB interventions program captured in this study were focused only on migrant workers and were delivered simultaneously with HIV interventions

### Interventions and program activities

Depending on type of KAPs, an HIV intervention provided a combination of services which may include 1) behavioral change program through outreach activities, harm reduction program (for PWID only), distribution of commodities such as condoms, lubricants, needles, syringes and behavior change communication (BCC) & IEC materials etc., 2) HIV counselling and testing through referral to health facilities, referral for STI testing, 3) referral to receive treatment, providing care & support for those tested positive, and following up on those on anti-retroviral therapy (ART) for adherence, and 4) community health system strengthening etc. TB interventions consisted of providing TB screening and testing services to migrant workers through outreach activities, distributing IEC materials for TB prevention and care, referring TB suspected cases to health facilities for further confirmational diagnosis, treatment, and care.

Community outreach activities and sessions were integral parts of HIV interventions in this study. They were often organized by outreach workers and peer educators who were recruited, trained, and supervised by IAs. Outreach sessions were often provided at a meeting venue in the community i.e., beauty salons, parks, stadium, universities, local markets, department stores, entertainment venue, public transportation stations, campaign events, beauty contests, and drop-in-centers (DiCs) run by IAs. KAPs were encouraged to get tested for HIV during outreach sessions and those who were willing to get tested were referred for voluntary HIV counselling and testing. HIV counselling and testing services were often provided at government health facilities such as the local hospital/ health service centers, mobile clinics, and community-based HIV testing centers. Outreach workers of IAs provided pre-test and post-test counselling, while HIV testing was performed by professional health staffs (nurses and medical technicians) using pin-prick blood testing with same day result (SDR).

### Program outputs

This study defined number of KAPs who received HIV services or population **reached **as key outputs yielded by intervention. Depending on intervention, this included KAPs who participated in outreach activities (i.e. individual sessions, group sessions, events, or contest etc.) organized by IAs and those who engaged in behavioral change programs through social media sessions carried out by IAs. MSM, TG, FSW and MSW who engaged in behavioral change program received IEC materials on HIV and STI prevention, and commodities such as condoms and lubricants. PWID engaged in harm-reduction programs received IEC materials on HIV and STI prevention, and clean needles & syringes. All of them underwent counseling service. Those who were willing to undertake HIV testing were referred to test at government health facilities. Those who requested or required STI testing were referred to health facilities to receive further services.

The term **‘tested’** as an output is defined as number of KAPs who were successfully referred and tested for HIV through an intervention. It is worth noting that HIV testing was often performed at government health facilities and cost were not counted towards those of the community outreach interventions. Therefore, this study did not examine unit cost per person tested.

For TB interventions, **reached** indicated number of migrant workers who underwent TB screening through outreach activities, and received IEC materials for TB prevention and care. The term ‘**tested’** for TB represented TB suspected cases found during screening and successfully referred for further confirmational diagnosis at government health facilities*.*

### Costing methodology

This study used a top-down costing approach and focused on health care providers perspective as interventions were implemented by IAs at the community level. Patients’ costs were not collected and included in this study. We collected data from expenditure records and progress update annual reports of IAs covering expenditures from 1st January 2015 to 30th September 2016. The data were keyed into excel sheets, pivotal tables were constructed to summarize and calculate unit cost. Shared costs between two or more interventions incurred by the same IAs were allocated proportionally by using different allocation strategies based on type of activities as they were described in the records or by matching with program outputs yielded by interventions.

### Direct program cost (IAs operational cost)

All cost incurred at site (provincial) level or IAs level were defined as direct program cost. According to IAs reports, costs were disaggregated into different level, firstly by program areas then by interventions, activities, and cost inputs. Shared cost across different KAPs or program area were allocated accordingly using allocation criterion based on their program outputs. Program cost included both recurrent and capital costs utilized by IAs which were reported into 13 cost inputs; human resources (HR), travel related costs (TRC), external professional services (EPS), health products - pharmaceutical products (HPPP), health products - non-pharmaceuticals (HPNP), health products - equipment (HPE) including HIV test kit, procurement and supply-chain management costs (PSM), infrastructure (INF), non-health equipment (NHE), communication material and publications (CMP), indirect and overhead costs, and living support to client/ target population (LSCTP).

### Indirect program cost (overhead cost)

Overhead costs were the cost incurred at higher administrative level by principal-subrecipient (PR) which included program management, health system strengthening, monitoring and evaluation etc. Principal-subrecipient (PR) received funding from donor and disbursed to IAs who were sub-recipients (SRs) for implementing programs. These costs were consumed by PR in Bangkok for overseeing the entire program throughout all provinces and all interventions. Some of these costs were not tied with a particular intervention or activities or geographical location. However, they need to be allocated reasonably and proportionally across all interventions. Therefore, overhead costs were calculated and allocated proportionally to all HIV interventions based on program outputs. We assumed that an intervention with higher program outputs consumed more overhead cost for program management.

### Analysis

In this study, we calculated two types of unit cost per population reached or persons receiving services from community outreach HIV interventions; 1) Average unit cost per person for each of 38 interventions, with inclusion of only program cost or operational cost of IAs incurred at site level while excluding overhead cost at above IAs level and 2) Average unit cost per person for 38 interventions, with inclusion of both program cost and overhead cost. Unit cost was defined as total cost divided by total program output. This study examined association between scale (quantity of population reached by intervention) and costs (unit cost per person) for interventions by applying bivariate regression forms available in MS Excel, which include linear, polynomial, and exponential. Unit of observations were the 38 interventions across provinces and KAPs. Costs versus scale was graphed with scatter plots, trendlines and coefficient of determination (R^2^) to portray range of results.

## Results

This study identified 38 community outreach HIV interventions implemented in 27 provinces of Thailand. Among these interventions, 14 targeted MSM/TG population, 12 were harm reduction interventions for PWID and their partners, 7 targeted migrant workers (MW), 4 targeted male sex workers (FSW) and 1 female sex workers (FSW). This study also pinpointed 7 TB interventions which were delivered in jointly with HIV interventions for migrant workers (Table 1).

### Program outputs

All interventions from 27 provinces in this study have successfully delivered HIV services to a total of 190,931 KAPs, 98,561 (51.6%) MSM/TG, 41636 (21.8%) MW, 25260 (13.2%) MSW, 13308 (7%) FSW, and 12,166 (6.4%) PWID. Interventions focused on sex workers (both female and male) reached most persons, with an average number of 7714 reached per intervention. Harm reduction interventions reached fewest persons, with an average number of 1014 reached per interventions which was six to seven times fewer than that of other interventions. Average number of persons reached per intervention for MSM/TG was 7040 and for migrant workers was 5948. Average number of populations reached, regardless of KAPs types, by an intervention was 5025 KAPs.

The HIV intervention for MSM/TG in Bangkok reached most people among 38 HIV interventions, having successfully delivered HIV services to 25,438 MSM/TG. Similarly, intervention focusing on migrant workers in Samut Sakhon, MSM/TG in Udon Thani, MSM/TG in Chiang Mai, and FSW in Bangkok reached most with 16,881 migrants, 14,528 MSM/TG, 13757 MSM/TG, and 13,308 respectively. The harm reduction intervention in Satun province, in the other hand, reached fewest with only 161 PWID (Table 2). The program of MSM/TG in Chiang Mai was the most successful intervention in terms of referrals for testing, by getting 4179 MSM/TG tested for HIV, followed by the program of MSM/TG in Bangkok with 4076 MSM/TG tested. Harm reduction programs were also least successful with fewer testing referrals (Table 2).

**Table 2:** Cost, outputs, and unit cost of HIV interventions in different provinces from January 2015 to September 2016

Provinces	Interventions	Cost (USD)			Outputs (Persons)		Unit Cost (USD per person)	
		Direct Program Cost	IPC (Overhead)	Total	Reached	Tested	Reached (Excl. IPC)	Reached (Incl. IPC)
Bangkok	FSW	294,562	130,376	424,937	13,308	1548	22.1	31.9
Chiang Mai	Migrants	191,020	92,533	283,553	5215	1228	36.6	54.4
Chon Buri	Migrants	75,779	35,454	111,233	2966	425	25.5	37.5
Rayong	Migrants	124,736	61,036	185,772	4740	970	26.3	39.2
Samut Prakan	Migrants	93,261	77,535	170,796	7369	743	12.7	23.2
Samut Sakhon	Migrants	182,912	180,886	363,798	16,881	1515	10.8	21.6
Songkhla	Migrants	152,667	33,718	186,385	2727	613	56.0	68.3
Trat	Migrants	58,144	18,842	76,985	1738	229	33.5	44.3
Bangkok	MSM/TG	444,216	294,597	738,813	25,438	4046	17.5	29.0
Chiang Mai	MSM/TG	204,023	178,388	382,411	13,757	4179	14.8	27.8
Chon Buri	MSM/TG	88,494	61,233	149,727	5599	744	15.8	26.7
Khon Kaen	MSM/TG	136,715	72,900	209,616	6480	1473	21.1	32.3
Nakhon Ratchasima	MSM/TG	101,277	57,128	158,406	5101	1079	19.9	31.1
Nakhon Si Thammarat	MSM/TG	162,830	82,147	244,977	7176	2176	22.7	34.1
Nonthaburi	MSM/TG	67,998	52,984	120,983	4542	539	15.0	26.6
Pathum Thani	MSM/TG	62,759	47,020	109,779	4138	709	15.2	26.5
Phuket	MSM/TG	68,036	43,753	111,788	3842	1017	17.7	29.1
Samut Prakan	MSM/TG	101,022	71,244	172,266	6206	643	16.3	27.8
Songkhla	MSM/TG	121,482	82,958	204,440	6845	936	17.7	29.9
Surat Thani	MSM/TG	71,528	26,204	97,732	2285	235	31.3	42.8
Ubon Ratchathani	MSM/TG	44,872	28,022	72,894	2430	391	18.5	30.0
Udon Thani	MSM/TG	100,294	55,541	155,835	4722	649	21.2	33.0
Bangkok	MSW	318,508	127,734	446,241	14,528	1435	21.9	30.7
Chiang Mai	MSW	103,077	32,039	135,115	3268	988	31.5	41.3
Chon Buri	MSW	147,453	76,362	223,815	6131	1242	24.1	36.5
Phuket	MSW	99,817	13,076	112,893	1333	470	74.9	84.7
Bangkok	PWID	382,652	41,909	424,561	2181	235	175.4	194.7
Chiang Mai	PWID	361,299	40,903	402,202	2276	535	158.7	176.7
Chiang Rai	PWID	92,040	9090	101,130	573	69	160.6	176.5
Narthiwat	PWID	346,993	48,496	395,488	2463	296	140.9	160.6
Pattani	PWID	102,864	12,187	115,051	655	129	157.0	175.7
Phatthalung	PWID	47,998	4886	52,884	270	45	177.8	195.9
Samut Prakan	PWID	80,086	4016	84,102	247	20	324.2	340.5
Satun	PWID	31,122	2765	33,887	161	67	193.3	210.5
Songkhla	PWID	111,541	16,614	128,155	803	166	138.9	159.6
Tak	PWID	233,059	24,564	257,623	1452	174	160.5	177.4
Trang	PWID	93,988	9903	103,891	565	317	166.3	183.9
Yala	PWID	74,972	12,102	87,074	520	34	144.2	167.4

*KAPs* key affected population, *IAs* implementing agencies, *IPC* indirect program cost, *Excl* exclude, *Incl* include, *FSW* female sex workers, *MSM/TG* men who have sex with men/ transgender, *MSW* male sex worker, *PWID* people who inject drug

### Cost of interventions

The total cost of 38 community outreach HIV interventions was 7,837,234 USD, over the period of 21 months from January 2015 to September 2016, which comprised direct program cost or operational cost of 5,576,092 USD (71.1%) consumed by implementing agencies (IAs) at site level for service delivery and indirect program cost or overhead cost of 2,261,142 USD (28.9%) incurred at above IAs level for program management and administration etc. (Table 1). The program cost of HIV interventions can be further divided into cost for 14 MSM/TG interventions (1,775,546 USD), 12 PWID interventions (1,958,613 USD), 7 MW interventions (878,518 USD), 4 MSW interventions (668,854 USD), and 1 FSW intervention (294,562 USD) respectively. Overhead cost of HIV interventions can be divided into cost for 14 MSM/TG interventions (1,154,119 USD), 12 PWID interventions (227,434 USD), 7 MW interventions (500,003 USD), 4 MSW interventions (918,065 USD), and 1 FSW intervention (130,376 USD), respectively (Table 3).

**Table 3:** Cost, inputs, outputs, and average unit cost of HIV interventions for different KAPs

Inter-vention	KAPs	No.	Inputs/Cost (USD)			Outputs (Persons)		Average unit cost (USD per person)	
			Program Cost	IPC (Overhead)	Total	Reached	Tested	Reached (Ex. IPC)	Reached (In. IPC)
HIV	MSM/TG	14	1,775,546	1,154,119	2,929,664	98,561	18,816	18.0	29.7
	MSW	4	668,854	249,211	918,065	25,260	4135	26.5	36.3
	FSW	1	294,562	130,376	424,937	13,308	1548	22.1	31.9
	PWID	12	1,958,613	227,434	2,186,047	12,166	2087	161.0	179.7
	Migrant	7	878,518	500,003	1,378,521	41,636	5723	21.1	33.1
	All^a^	38	5,576,092	2,261,141	7,837,234	190,931	32,309	29.2	41.0
TB	Migrant^b^	7	1,110,065	526,017	1,636,082	56,099	878	19.8	29.2
HIV/TB	Migrant^c^	14	1,988,583	1,026,020	3,014,603	97,735	6601	20.3	30.8

^a^All HIV interventions regardless of KAPs group

^b^TB interventions for migrant workers

^c^Interventions (TB/HIV) focus on migrant workers

*KAPs* key affected populations, *IPC* indirect program cost, *Ex* exclude, *In* include, *FSW* female sex workers, *MSM/TG* men who have sex with men/ transgender, *MSW* male sex worker, *PWID* people who inject drug

The cost of HIV intervention for MSM/TG in Bangkok was the highest among all interventions at 738813 USD (with program and overhead cost combined) followed by MSW in Bangkok (446,241 USD), FSW in Bangkok (424,937 USD), PWID in Bangkok (424,561 USD) and harm reduction program in Chiang Mai (402,202 USD) respectively. The cost for the harm reduction program in Satun was the lowest by only 33,887 USD.

For TB interventions, the total cost of 7 interventions was 1,636,082 USD, which can be breakdown into direct program cost of 1,110,065 USD (67.8%) and indirect program cost or overhead cost of 526,017 USD (22.2%) (Table 2).

### Unit cost per person

Average unit cost per person reached or received HIV services, without inclusion of overhead cost, was 18 USD for MSM/TG, 21.1 USD for MW, 22.1 USD for FSW, 26.5 USD for MSW, and 161 USD for PWID (Table 3). The results show that HIV intervention for migrants in Samut Sakhon province was found to be the least costly with the lowest unit cost of 10.8 USD per person reached, which is followed by intervention for migrants in Samut Prakan at 12.7 USD per person reached, MSM/TG in Chiang Mai (14.8 USD per person reached), MSM/TG in Nonthaburi (15 USD) and MSM/TG (15.2 USD) in Pratum Thani (Table 2). Harm reduction program in Samut Prakan recorded the highest unit cost of 324.5 USD per person to received services (Table 2).

After inclusion of overhead cost into the calculation, average unit cost per person reached was 29.7 USD for MSM/TG, 33.1 USD for MW, 31.9 USD for FSW, 36.3 USD for MSW, and 179.1 USD for PWID (Table 3). The intervention for migrants in Samut Sakhon remained as the least costly despite a nearly 100% increase after adding overhead cost. This is followed by intervention for migrants in Samut Prakan (23.2 USD per person reached), MSM/TG (26.5USD) in Pratum Thani, MSM/TG in Nonthaburi (26.6 USD) and, MSM/TG in Chon Buri (26.7 USD) (Table 2). Harm reduction in Samut Prakan remained the intervention with the highest unit cost of 340.5 USD per person reached, although there was only a small 5% increase after adding overhead cost (Table 2). For TB interventions, the average unit cost per person reached or screened for TB was 19.8 USD without inclusion of overhead cost and 29.2 USD with.

### Economies of scale and scope

**Fig. 2:**
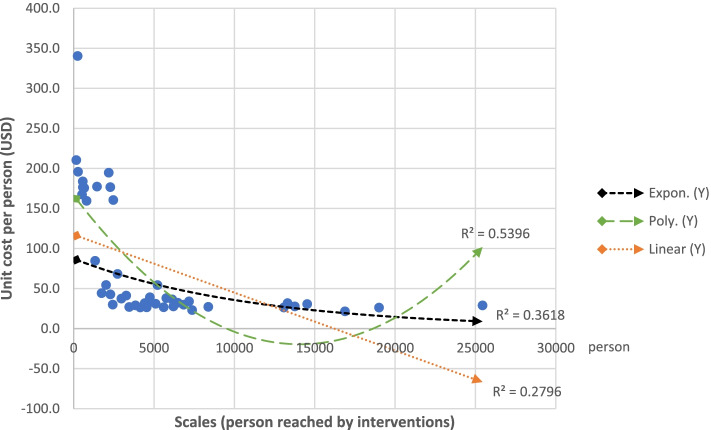
Scales vs unit cost per person of all interventions with linear, exponential, and polynomial trend line

**Fig. 3:**
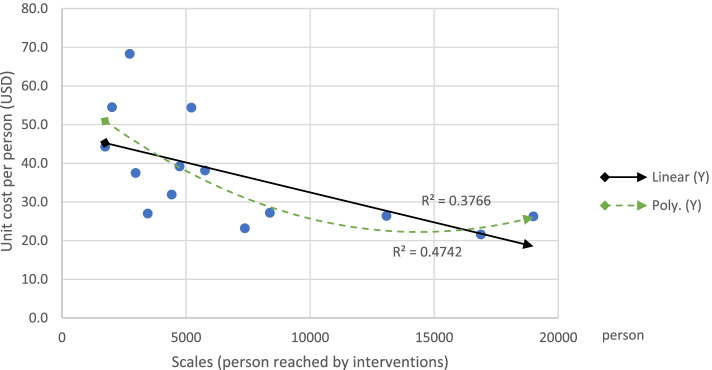
Scales vs unit cost per person of HIV and TB interventions for migrants

We found that, overall, there was a negative association between scale and unit cost of interventions in terms of persons reached. The coefficient estimate for covariate was − 0.007 indicating that for every additional person reached, unit costs decreased by 0.007 USD. This finding is statistically significant (*p* = 0.0002, Table 4). It indicates that efficiency of intervention increased (unit costs decreased) with scale. The regression line is downward sloping as shown in Fig. 2 and suggests that there was an up-turn point observed when fitting a polynomial trend line, however, this is merely due to one data point among all interventions. The findings suggest that there are economies of scale in the provision of HIV intervention. Similarly, there was a statistically significant negative association between scale and unit costs for HIV and TB interventions for migrants, (Fig. 3, and Table 4). The estimate coefficient for covariate was − 0.002 suggesting that an additional person reached, unit costs decreased by 0.002 USD (*p* = 0.02, Table 4). The average unit cost per person for a migrant for a combined HIV/TB intervention was 20.3 USD (without overhead cost), which is lower than the summed-up unit costs for separate HIV (21.1 USD) and TB interventions (19.8 USD, Table 3). This suggests that there may be economies of scopes in the joint provision of HIV/TB interventions for migrants. In other words, implementing HIV and TB interventions in combination may lead to a reduction in unit costs, compared to separate delivery.

**Table 4:** Estimated coefficient between scale and unit cost of interventions with linear regression

Interventions	No	Coefficient	R^**2**^	***P***-value
All interventions (HIV/TB)	45	−0.007	0.2796	0.0002
Migrants (HIV/TB)	14	−0.002	0.3766	0.0196
MSM/TG (HIV)	14	−0.000	0.0516	0.4348
PWID (HIV)	12	−0.021	0.1323	0.2452

Interventions for MSW and FSW were excluded in regression analysis due to small sample size and insignificant *P*-value

## Discussion

Thailand has achieved its first and third goals of UNAIDS’ 90–90-90 targets in 2018, with more than 90% of people living with HIV in Thailand aware of their status and 80% of them on treatment thanks to successful HIV prevention, testing and treatment programs [1]. Since Thailand’s HIV epidemic is concentrated in KAPs, this study demonstrates the approach of targeting KAPs in priority geographical sites is both feasible and effective. The results of this study show that community outreached interventions led by NGOs and CSOs are critical in Thailand’s new approach for fighting against the epidemic. These interventions delivered HIV and TB services to many KAPs and linked them into HIV prevention and life-long treatment under the RRTTR program.

We found that costs of HIV interventions vary across targeted population types and geographical areas in Thailand. Average unit cost per person of an intervention for MSM/TG was the lowest followed by interventions for MW, MSW, FSW, and PWID, respectively. In Thailand, HIV prevention services led by NGOs for MSM/TG and other KAPs were available countrywide prior to the RRTTR program, especially in large cities and tourist destinations where MSM/TGs were concentrated [2, 5, 10, 11]. Some IAs were, therefore, were able to integrate the RRTTR programs into their existing programs rather than creating new implementing structures and plans. As a result, this might have reduced start-up cost and reduced costs of interventions [7]. In addition, not only are the MSW, FSW, and PWID populations relatively smaller than MSM/TG and MW, they are also harder to reach as many of them are often reluctant to open their identity to others [12]. Average unit cost of an intervention for PWID was found five folds higher than other HIV interventions (Table 3). Low uptake of harm reduction intervention services for PWID found in this study suggested that they were less viable compared to interventions focusing on other KAPs. There are existing studies indicating that policy and legal constraints have worsened access to lifesaving healthcare services for PWID in Thailand. Fear of disclosure, stigma and discrimination in healthcare settings and concerns over confidentiality were among the main factors which causes low demand for HIV services for PWID [12–14]. Therefore, utilization of harm reduction services was often low and more intensive service delivery with comprehensive package was needed to reach the PWID population. HIV interventions for migrants in Samut Prakan and Samut Sakhon were the least costly, with the lowest unit cost among all. We found evidence of economies of scope in joint HIV and TB interventions for interventions targeted at migrants in this study. Under the RRTTR program, IAs delivering services for migrants were granted funding support for implementing both HIV and TB interventions. It allowed IAs to share available resource between the two programs, sharing fixed cost, intensifying demand-creation activities, and expanding existing programs rather than creating new structures for each program [7]. Sharing of services can reduce costs, and task shifting can lead to efficiency gains through integrated services [7, 15]. However, there are many contextual factors to be considered when interpreting costs of HIV interventions. This includes IAs’ program maturity, geographic structural factors (i.e., difficulty of access in remote locations), quality of services provided and efficiency of service delivery etc. Program immaturity is likely associated with high start-up cost for new service or location and insufficient service delivery processes [7]. And higher quality services with more comprehensive care packages are likely to result in higher unit cost and lower numbers reached [16, 17].

In general, interventions are more likely to achieve higher efficiency in large metropolitan and surrounding areas such as Bangkok, Chiang Mai, Chonburi, Samut Prakarn and Samut Sakhorn. In many provincial regions, in contrast, low uptake of services was observed, resulting in higher unit cost for interventions. In addition, harm reduction programs were found more costly compared to interventions focusing on other KAPs. This study found that unit costs of HIV interventions led by community based IAs decreased with scale. This suggests that there are economies of scale, as unit cost per key outputs and scale are found to be correlated negatively (Fig. 2). Therefore, this study shows that while the overall costs of community outreach HIV and TB interventions increase when being scaled up, the unit cost per person will decrease as more persons are being reached [8, 18].

This research study is subject to some potential limitations that could be addressed in future studies. It is worth noted that the method used for overhead cost allocation in this study may overestimate costs for interventions with high outputs and have an impact on efficiency analysis. Initially, interventions under this program were funded for 2 years. However, an extension for another 2 years was granted by the Global Fund when it ended. At the time of data collection for this study, we were only able to collect data covering the initial phase of the program. Therefore, a further analysis should be conducted with data on the secondary phase. This study applies only cost of HIV services provided at the community level which does not include cost incurred from patients’ perspective. And future studies should focus on investigating other potential contextual factors that might act as cost drivers for interventions and discussing whether the same results, or better results might have been achieved with changes to the way interventions are implemented. In addition, other components of RRTTR (i.e., testing and provision of treatment under conventional public health system etc.) should also be evaluated in future studies.

## Conclusion

This study found that the average unit costs of HIV interventions vary across targeted populations and geographical areas in Thailand. The interventions were more likely to achieve lower unit cost if they were implemented in large metropolitan and surrounding areas. Harm reduction program was the costliest compared with other types of intervention. Overall, there was evidence of economies of scale suggesting that the average unit costs of community outreach HIV and TB interventions led by CSOs will decrease as they are scaled up. There was also evidence of economies of scope indicating that joint provision of HIV prevention and TB services reduced unit cost compared to separate provision. Further studies are suggested to follow up with these ongoing interventions for identifying potential contextual factors to improve efficiency of HIV prevention services in Thailand.

## Data Availability

The datasets used and/or analyzed during the current study are available from the corresponding author on reasonable request.

## Abbreviations

HIV: Human immuno-deficiency virus
ART: Anti-retroviral therapy
RRTTR: Reach-Recruit-Test-Treat-Retain program
NGOs: Non-governmental organizations, CSOs civil society organizations
KAPs: Key-affected populations
MSM/TG: Men who have sex with men/ transgender
MSW: Male sex worker
FSW: Female sex worker
PWID: People who inject drug
MW: Migrant workers
IAs: Implementing agencies
DiCs: Drop-in-centers
VCCT: Voluntary confidential counselling and testing
STI: Sexual transmitted diseases
PR: Principal recipient
SRs: Sub-recipients
TB: Tuberculosis

## References

[1] The Joint United Nations Programme on HIV/AIDS (UNAIDS) (2019). AIDS data.

[2] National AIDS Committee Thailand (NAC) (2014). Thailand AIDS Response Progress Report.

[3] Patcharanarumol W, Thammatacharee N, Kittidilokkul S (2013). Thailand’s HIV/AIDS program after weaning-off the global fund’s support. BMC Public Health.

[4] The Joint United Nations Programme on HIV/AIDS (UNAIDS) (2018). Country Snapshot, Thailand.

[5] National AIDS Committee Thailand (NAC). Thailand National Operational Plan 2015–2019. Nonthaburi: National AIDS Management Center, Department of Disease Control, Ministry of Public Health, Thailand; 2014. https://hivhub.ddc.moph.go.th/Download/Strategy/EN2_Thailand%20National%20Operational%20Plan%20Accelerating%20Ending%20AIDS_2015-2019.pdf.

[6] The Global Fund (2015). Grant Agreement on Stop TB and AIDS through RRTTR (STAR) Thailand.

[7] Mariana S, Michelle R, Carol DO, Claudia BM, Karl LD, Anna V (2014). Is there scope for cost savings and efficiency gains in HIV services? A systematic review of the evidence from low- and middle-income countries. Bull World Health Organ.

[8] Carol DO, Lorna G, Sedona S, Integra I, Anna V (2016). Does integration of HIV and SRH services achieve economies of scale and scope in practice? A cost function analysis of the integra initiative. Sex Transm Infect.

[9] Galárraga O, Wirtz VJ, Figueroa-Lara A, Santa-Ana-Tellez Y, Coulibaly I, Viisainen K (2011). Unit costs for delivery of antiretroviral treatment and prevention of mother-to-child transmission of HIV: a systematic review for low- and middle-income countries. Pharmacoeconomics.

[10] Wolf RC (2012). Thailand global fund round 8 external evaluation: men who have sex with men (MSM).

[11] Taweesap S, Sumet O, Patchara B, Wiwat P, Sharma M (2016). The impact of Thailand's public health response to the HIV epidemic 1984–2015: understanding the ingredients of success. J Virus Erad.

[12] Churcher S (2013). Stigma related to HIV and AIDS as a barrier to accessing health care in Thailand: a review of recent literature. WHO South East Asia J Public Health.

[13] Ti L, Kaplan K, Hayashi K, Suwannawong P, Wood E, Kerr T (2013). Low rates of hepatitis C testing among people who inject drugs in Thailand: implications for peer-based interventions. J Public Health.

[14] Kanna H, Will S, Joanne C, Sattara H, Thomas K. Experiences with policing among people who inject drugs in Bangkok, Thailand: a qualitative study. Plos Med. 2013;10(12). 10.1371/journal.pmed.1001570. 10.1371/journal.pmed.1001570 PMC3858231 24339753

[15] Gr O, Richard G, Sandra GSR, Mercy GM, David CL, Sergio BA (2017). HIV prevention costs and their predictors: evidence from the ORPHEA project in Kenya. Health Policy Plan.

[16] Chandrashekar S. Scaling up HIV/AIDS prevention in India: an economic analysis of Avahan interventions for high-risk groups in four southern states: MPhil thesis, London School of Hygiene & Tropical Medicine; 2015. 10.17037/PUBS.02997235.

[17] Sergio BA, Gina LHF, David CL, Ada K, VB SJ, Ogbonna OA, et al. Efficiency of HIV services in Nigeria: Determinants of unit cost variation of HIV counseling and testing and prevention of mother-to-child transmission interventions. Plos One. 2018;13(9). 10.1371/journal.pone.0201706. 10.1371/journal.pone.0201706 PMC6128456 30192765

[18] Marseille E, Dandona L, Marshall N (2007). HIV prevention costs and program scale: data from the PANCEA project in five low and middle-income countries. BMC Health Serv Res.

