# Correction: The cost of community outreach HIV interventions: a case study in Thailand

**DOI:** 10.1186/s12889-026-27670-0

**Published:** 2026-05-25

**Authors:** Kyaw Min Soe, Katharina Hauck, Sukhum Jiamton, Sukhontha Kongsin

**Affiliations:** 1 Research Centre for Health Economics and Evaluation, Faculty of Public Health, Mahidol University, 420/1 Ratchawithi Rd, Khet Ratchathewi, Bangkok, 10400 Thailand; 2 Department of Infectious Disease Epidemiology, Faculty of Medicine, School of Public Health, Imperial College London, London, UK; 3 Department of Dermatology, Faculty of Medicine Siriraj Hospital, Mahidol University, Bangkok, Thailand

**Correction: BMC Public Health 22**,** 20 (2022)**

**https://doi.org/10.1186/s12889-021-12416-x**

Following publication of the original article [1], the authors reported an error under Ethics approval and consent to participate section. The updated Ethics approval and consent to participate is given below and the changes have been highlighted in **bold typeface**.

**Ethics approval and consent to participate**

The study applied secondary data collected by Research Centre for Health Economics and Evaluation, Faculty of Public Health, Mahidol University which was used for the evaluation of RRTTR program. It was approved by Ethical Reviewing Committee, Faculty of Public Health, Mahidol University, Thailand (**COA # MUPH 2017-172**). All procedures were performed in accordance with relevant guidelines. Every precaution was taken to protect the privacy of research subjects and the confidentiality of their personal information.

The original article [1] has been updated.
